# Bio‐Revitalizing SkinGlow: Assessing the Efficacy of Microcannula‐Assisted Treatment With Cohesive Polydensified Matrix Hyaluronic Acid With Glycerol (Belotero Revive) Through Ultrasound Elastography and Corneometry

**DOI:** 10.1111/jocd.70509

**Published:** 2025-10-17

**Authors:** Suhail Luna

**Affiliations:** ^1^ Xiluet Medical Private Practice Guadalajara Mexico

**Keywords:** Belotero Revive, cannula, cohesive polydensified matrix, corneometry, CPM‐R, hyaluronic acid, skin hydration, strain elastography

## Abstract

**Introduction:**

Despite the widespread use of hyaluronic acid (HA)‐based fillers, most studies have focused on volumetric restoration rather than skin quality improvement. Objective imaging techniques, such as ultrasound strain elastography and corneometry, allow quantitative and qualitative assessment of treatment efficacy, filling an important gap in aesthetic dermatology research.

**Objective:**

To evaluate the safety and efficacy of treatment using the cohesive polydensified matrix hyaluronic acid with glycerol (CPM‐R, Belotero Revive; Anteis, Plan‐les‐Quates, Switzerland) delivered via microcannula for skin hydration, firmness, and elasticity.

**Methods:**

A retrospective cohort study was conducted, with eligible patients divided into 2 groups: patients who had received a single (group 1) or a two‐session treatment (group 2) of CPM‐R. Patients were evaluated at baseline and 30 days after the final treatment by corneometry and ultrasound strain elastography. At the final follow‐up appointment (Day 30 (D30) for Group 1, Day 60 (D60) for Group 2), both the investigator and the patient assessed outcome efficacy using the 5‐point Global Aesthetic Improvement Scale (GAIS) and the Pain and Comfort Visual Analogue Scales (VAS).

**Results:**

A total of 40 patients were included in the final analysis; mean age was 41.7 years (range: 25–60), 85.0% were female (*n* = 34/40), and most patients had mild rosacea, acne‐prone, or dry skin. Of the 40 patients, 6 (16.0%) exhibited mild rosacea, and 12 (30.0%) had acne‐prone skin. No patients withdrew from the study. Hydration levels, as assessed by corneometry, significantly improved following treatment with CPM‐R by an average of 14.9% after a single treatment and 16.6% after two treatments spaced four weeks apart (*p* < 0.001). The investigator and patient evaluations of facial appearance were either “much improved” or “very much improved” in all patients who underwent a single‐treatment or two‐treatment protocol. No serious adverse events were reported. Minor and transient side effects (e.g., local mild erythema and ecchymosis) were reported in 15.0% (*n* = 6/40) and 7.5% (*n* = 3/40) of cases, respectively, and resolved spontaneously within 24–72 h. Patients reported minimal discomfort, with 75.0% (*n* = 30/40) of the patients reporting an average VAS pain score of 1 (out of 10).

**Conclusions:**

This study demonstrated that the use of CPM‐R delivered through retro‐linear and fanning techniques with a microcannula is an innovative, effective, safe, and well‐tolerated intervention for improving skin hydration and overall dermal quality in patients with mild to moderate signs of aging.

## 1 Introduction

Skin aging is a multifactorial process influenced by both intrinsic (e.g., genetics, hormonal decline) and extrinsic (e.g., ultraviolet exposure, pollution, lifestyle) factors, and may be clinically translated as dehydration, loss of elasticity, and appearance of visible signs such as wrinkles and fine lines [1].

Hyaluronic acid (HA) is a biocompatible glycosaminoglycan ubiquitous in the human body, especially in the extracellular matrix (ECM), playing a paramount role in tissue hydration and cellular processes such as proliferation and differentiation [2]. Thus, there is a growing interest in therapeutic endeavors, including minimally injectable HA fillers for aesthetic purposes. Although traditional injectable HA formulations primarily target facial volume loss, newer formulations, such as Cohesive polydensified matrix HA (CPM‐HA) with glycerol (CPM‐R, Belotero Revive; Anteis S.A., Plan‐les‐Ouates, Switzerland), focus on skin hydration, elasticity, and dermal quality improvement rather than volumization [3]. The combination of HA and glycerol in CPM‐R enhances its potential for skin hydration by creating a moisture‐retaining reservoir in the ECM while improving collagen organization and viscoelastic properties. Furthermore, objective imaging approaches such as ultrasound strain elastography and corneometry allow quantitative and qualitative assessment of treatment efficacy, filling an important gap in aesthetic dermatology research.

The use of a microcannula as the delivery instrument in this study was a deliberate choice aimed at optimizing both patient comfort and treatment safety. Compared to traditional needles, microcannulas significantly reduce tissue trauma by gliding through anatomical planes rather than piercing blood vessels and nerve endings, which results in less bruising, swelling, and discomfort. This research aims to evaluate hydration and elasticity of CPM‐R using objective measurements, as well as evaluate comfort, safety, and recovery time with the use of the microcannula technique.

## 2 Materials and Methods

### 2.1 Study Design

A retrospective cohort study was conducted in accordance with the 1964 Helsinki declaration and its later amendments or comparable ethical standards, and a centralized institutional review board approval was obtained. All patients provided written informed consent and photographic informed consent.

### 2.2 Objectives

The primary study objective was to assess the efficacy and safety of CPM‐R treatment via microcannula delivery (The SkinGlow technique) in improving skin hydration, elasticity, and overall dermal quality as measured via corneometry and strain elastography (SE).

Secondary objectives were:

1. Comparison of single versus two‐session treatments in terms of skin hydration and elasticity.
2. Evaluation of the pain and patient procedural comfort perception with microcannula‐assisted HA delivery.
3. Safety of the microcannula‐assisted HA delivery of CPM‐R.
4. Impact of CPM‐R in rosacea and acne‐prone skin and evaluation additional benefits of glycerol in skin barrier function.

Patients from a single private aesthetic medicine clinic in Guadalajara, Mexico were selected from April 2024 to December 2024, based on the availability of the following criteria: adults aged 25–60 years, Fitzpatrick Skin Types II–IV, with mild‐to‐moderate signs of dry skin, who had been submitted to treatment with CPM‐R for improving skin revitalization without volume augmentation (either with 1 or 2 treatments), and availability of pre‐treatment and 30‐day post‐treatment assessments using corneometry and elastography. Patients with a medical history of hypersensitivity to HA or glycerol, pregnancy or breastfeeding, presence of active skin infections or inflammatory conditions at the injection site, and previous aesthetic treatments (HA fillers, energy‐based devices) within the past 4 months of the treatment were excluded.

Eligible participants were divided into two groups based on the clinical evaluation of skin hydration:

- Group 1: patients with low‐to‐moderate dry skin who had received 1 treatment with CPM‐R (each treatment with 3 syringes, being 1.5 syringes per side).
- Group 2: patients with severe dry skin who had received 2 treatments of CPM‐R (each treatment with 3 syringes, being 1.5 syringes per side. Treatments were performed 4 weeks apart).

All patients were followed up until 30 days after the last injection (Day 30 (D30) for group 1 and Day 60 (D60) for group 2). Objective assessments (Corneometry and strain elastography) were performed before treatment (baseline) and 30 days after each injection, whereas investigator and patient assessments were performed at the end of the treatment (D30 for group 1 and D60 for group 2). The sample consisted of patients selected by convenience and did not include a formal sample size calculation.

### 2.3 Assessments

#### 2.3.1 Corneometry and Strain Elastography

Stratum corneum hydration was assessed by a capacitance device (corneometry; CM 825, COURAGE+ KHAZAKA) and elasticity by ultrasound strain elastography (L20 HD3 Ultrasound, Clarius Mobile Health, portable L20 HD3 linear probe 20 MHz). Corneometry measurements were conducted under standardized conditions, with patients instructed to avoid applying any topical skincare products for at least 12 h prior to the evaluation to minimize confounding factors. Elastography refers to ultrasound‐based techniques that assess tissue stiffness or elasticity. Two primary modalities include strain elastography (SE) and shear wave elastography (SWE). While SE measures relative tissue deformation in response to manual compression, SWE quantitatively evaluates the speed of shear wave propagation within tissue, offering greater reproducibility and sensitivity to mechanical differences. In SE, the tissue is compressed with the ultrasonographic transducer, the acquired images are digitally processed, and the qualitative results are presented as a color‐coded map, in which tissues with various stiffness are qualitatively classified by different colors (more elastic tissues (softer tissues) are highlighted in red, whereas intermediate elasticity and low elasticity (stiffer tissue) are highlighted in green and blue, respectively) [4]. A standardized 5‐point visual scoring system was used to classify tissue elasticity: Score 1 (very soft), Score 2 (soft), Score 3 (intermediate), Score 4 (firm), and Score 5 (very firm).

Assessments were performed at baseline and 30 days after each injection.

#### 2.3.2 Investigator Assessment and Patient Reported Outcomes

Clinical assessment at baseline included evaluation of the presence of dry skin only, rosacea (erythema and/or telangiectasia), or acne‐prone skin (roughness, comedones and/or imperfections).

Treatment was assessed by the investigator and by patients at the end of the treatment (D30 for group 1 and D60 for group 2) using a 5‐point Global Aesthetic Improvement Scale (GAIS) (“worse, −1”; “no change, 0”; “improved, 1”; “much improved, 2”; and “very much improved, 3”) as compared to baseline. Moreover, patients were required to answer a 4‐point multiple choice question inquiring about their “Overall patient satisfaction with the treatment” (by selecting one of: Very Satisfied, Satisfied, Neutral, Dissatisfied) to evaluate their holistic experience throughout the treatment. Additionally, at the end of the treatment (D30 for group 1 and D60 for group 2), patients were asked “What is the most improved aspect of your treatment?” in their opinion, and to score pain during the procedure using the Pain and Comfort visual Analogue Score (VAS; 0 as “no pain” on the scale to 10 as “worst pain”) [5]. Adverse events (AEs) that were reported by the patient or observed by the investigator were recorded on a continuous basis throughout the trial.

### 2.4 Technique Description

**FIGURE 1:**
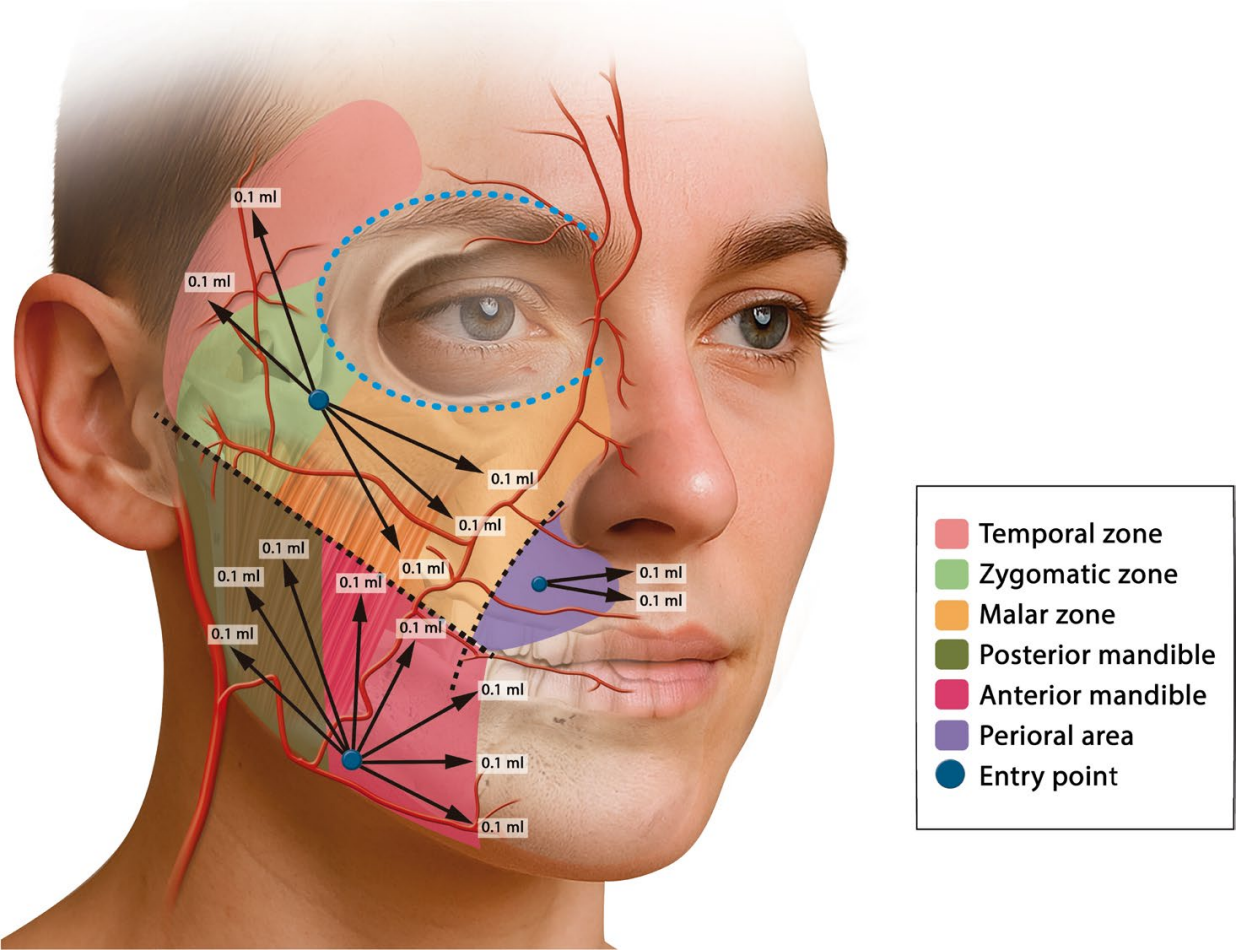
Schematic representation of the SkinGlow technique showing the entry points (blue circles), volume of product delivered and direction of retrograde injections (white arrows). The technique involves three entry points; one at the zygomatic area which covers hydration to temporal, zygomatic, malar, and periocular (black dashed line around the eye) zones. The second at the mandibular zone (anterior and posterior mandible) and the third one at the perioral region which is located 2 cm anterior to the nasolabial fold (green dashed line) and 1 cm superior to the oral commissure. Retrograde injections are performed using a 22G × 50 mm cannula delivering 0.1 mL of CPM‐R evenly distributed along the subcutaneous plane to ensure homogeneous integration. Black dashed line represents the line from the oral commissure to the tragus as a reference. Courtesy of Merz.

The “SkinGlow” technique (Figure 1) was designed to improve dermal quality in patients presenting early signs of aging, chronic dry skin, irregular texture, or tone changes—without inducing volume, altering facial structure, causing inflammation, or generating discomfort.

First, a thorough cleansing, followed by facial marking identifying three anatomical points per hemiface, is performed as follows:

1. Zygomatic entry point: Located at the level of the zygomatic ligament (middle third of the face). This entry point covers hydration to malar, zygomatic, periocular and temporal zones. Using the line from the oral commissure to the tragus as a reference, care should be taken to ensure that the cannula reaches up to 1 cm anterior to the tragus to avoid compromising the temporal arteries. Although the product is administered superficially in the subcutaneous plane, this precaution is taken to minimize the risk of vascular complications.
2. Mandibular entry point: Located at 1 cm of the anterior border of the masseter muscle allows approaching of the inferior zone of the face and the mandibular angle.
3. Perioral entry point: In case of treating fine lines (perioral/barcode lines) in the patient, this region is optional since not all the patients present dry skin or aging signs in this area. This entry point is located 2 cm anterior to the nasolabial fold and 1 cm superior to the oral commissure. In this case, retro‐injections can be performed on one side or bilaterally.

Through retro‐injection and fanning techniques, 0.5 mL should be injected in the zygomatic area, 0.8 mL in the mandibular area, and 0.2 mL in the perioral areas (0.1 mL per linear thread) in the subcutaneous plane.

The radial distribution guarantees a homogeneous coverage, following a divergent lines pattern from each entry point.

A total of 3 mL of CPM‐R (3 syringes; 1.5 mL/side) should be administered per session, intended for skin revitalization rather than volume enhancement. A gentle massage to ensure an even distribution of the product, avoiding boluses and superficial irregularities, should be performed.

For the post‐treatment care, it was recommended to refrain from strenuous exercise, excessive sweating, or intense heat exposure (e.g., saunas, hot tubs) for 48 h. Patients were instructed to avoid touching or massaging the treated area, applying make‐up, and consumption of alcohol or anti‐inflammatory medications (e.g., ibuprofen, aspirin) unless prescribed, as these can increase bruising. Moreover, the use of exfoliating products, retinoids, vitamin C serums, AHAs/BHAs, and peelings should be avoided for 5–7 days. Applying cold compresses for the first 24 h can improve swelling, and the use of a gentle, non‐comedogenic cleanser, fragrance‐free moisturizer, and broad‐spectrum sunscreen (SPF 50+) daily for at least 7 days post‐treatment should be performed.

### 2.5 Statistical Analysis

Categorical variables were described using frequencies (*n*) and percentages (%), while continuous variables were described using mean values and standard deviations. Linear mixed‐effects models were used to assess improvement in hydration over time between groups. These models include patients as a random effect and group, time, and their interaction as predictors. The results are presented as means with a 95% confidence interval (95% CI) and *p*‐values. Comparisons between groups 1 and 2 for GAIS patients, GAIS investigators, VAS pain scores, overall satisfaction, and the presence of AEs were evaluated using the Pearson chi‐squared test or Fisher's exact test. The adverse events were described by group. All these comparisons were then analyzed by stratifying for rosacea, dry skin, and acne‐prone skin. The statistical analysis was performed using StataNow/MP 19.5 (StataCorp, College Station, TX, USA). All tests were two‐tailed, being considered significant if *p* < 0.05.

## 3 Results

### 3.1 Demography

The study included 40 patients (*n* = 20 in each group), with a mean age of 41.7 years (range: 25–60). Patients were predominantly female (85.0%, *n* = 34/40). Presence of dry skin alone (57.5%, *n* = 23/40) was most frequently reported, followed by acne‐prone skin (30.0%, *n* = 12/40) and mild rosacea (15.0%, *n* = 6/40). The demographic baseline was not similar between the 2 groups since it was a convenience sample.

### 3.2 Skin Hydration (Corneometry Results)

**FIGURE 2:**
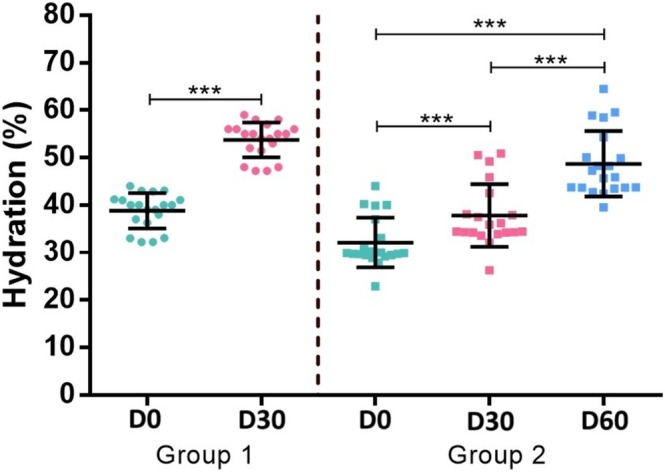
Mean hydration levels comparing both groups. Baseline hydration levels of group 2 were significantly lower than group 1, aligned with the clinical evaluation of severe dry skin. Both groups presented significant improvement in hydration compared to baseline at the end of follow‐up (30 days after the last injection). For group 2, a cumulative effect was perceived since hydration levels were significantly greater after the 2nd injection, indicating a sustained effect of hydration, as well as a booster effect. (***p < 0.001).

Hydration levels at baseline for group 2 were significantly lower than for group 1 (*p* < 0.001; Table 1, Figure 2). Hydration significantly improved following treatment with CPM‐R, with mean hydration values at end of the follow‐up of 53.7% ± 3.7% after a single treatment and 48.7% ± 6.9% after two treatments spaced four weeks apart (*p* < 0.001). Within group 2, the mean increase in hydration levels compared to baseline was 5.7% ± 0.5% (95% CI: 4.8%–6.6%; *p* < 0.001) at D30 and 16.6% ± 0.5% (95% CI: 15.7%–17.5%; *p* < 0.001) at D60.

**TABLE 1:** Hydration levels as per corneometry for both groups.

Hydration (%)	Baseline	End of follow‐up	*p*
Group 1 (*n* = 20)	38.8 ± 3.7	53.7 ± 3.7	< 0.001
Group 2 (*n* = 20)	32.1 ± 5.3	48.7 ± 6.9	< 0.001
*p*‐Value	< 0.001	0.002	

*Note:* Mean values and standard deviation at baseline and end of follow‐up (30 days after last injection). Group 1: patients with low‐to‐moderate dry skin who had received 1 treatment with CPM‐R. Group 2: patients with severe dry skin who had received 2 treatments of CPM‐R.

Mean absolute post‐treatment hydration levels at the end of follow‐up were significantly greater in the single treatment group (53.7% ± 3.7%) compared to the 2‐treatment group (48.7% ± 6.9%; *p* = 0.002). However, baseline hydration levels were significantly different between both groups (*p* < 0.001; Table 1). Nonetheless, in the adjusted model, mean increase in hydration levels compared to baseline was significantly higher for group 2 (16.6%; 95% CI: 15.8%–17.4%) compared to group 1 (14.9%; 95% CI: 14.2%–15.7%; *p* = 0.003; Figure 2).

### 3.3 Tissue Elasticity (Strain Elastography Results)

**FIGURE 3:**
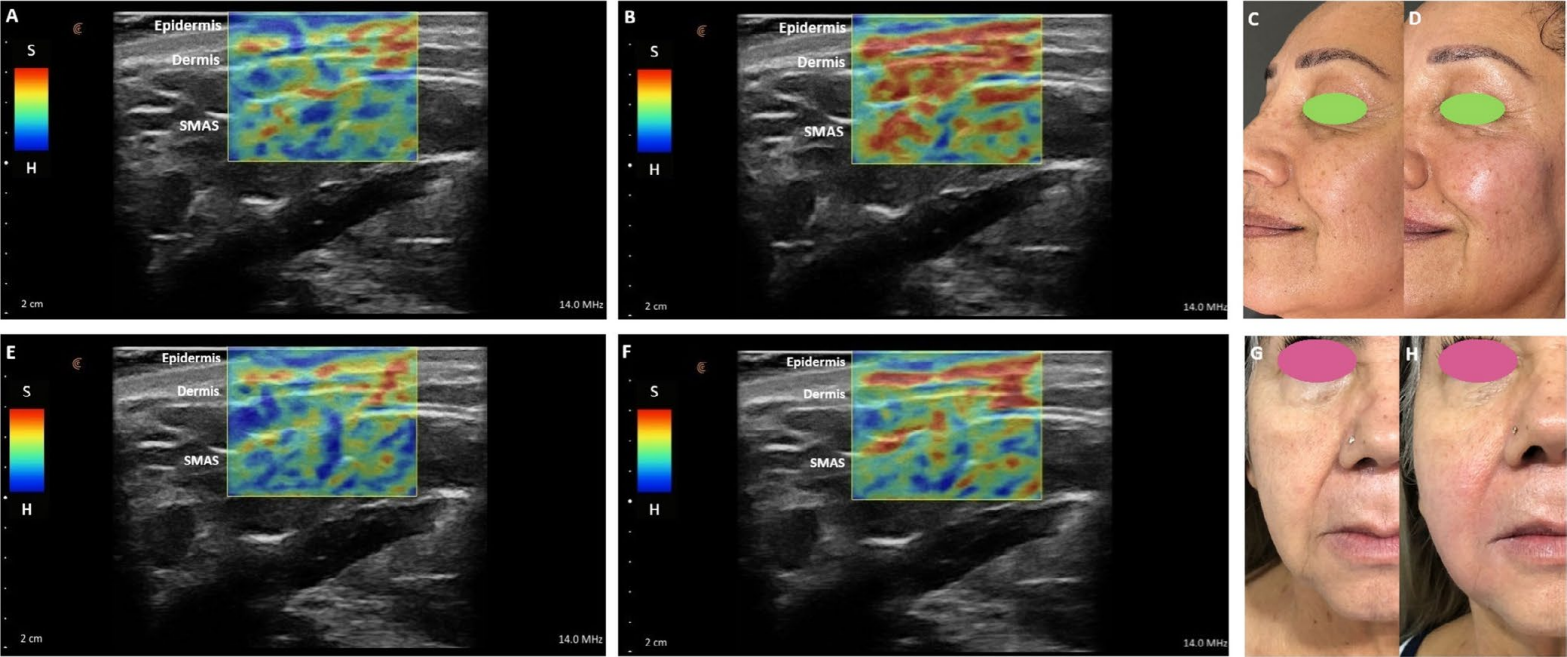
Ultrasound strain elastography and clinical images of two female patients before and after treatment with CPM‐R via the SkinGlow technique, showing improvement in tissue elasticity and firmness. Panels A‐D correspond to a 55‐year‐old female patient who received one session of the SkinGlow technique, with skin hydration increasing from 36.3% to 51.5%. Elastography images at baseline (A) and 30 post‐treatment (B); clinical image before (C) and after treatment (D). Panels E–H correspond to a 60‐year‐old female patient who received 2 sessions of the SkinGlow technique, with skin hydration increasing from 22.9% (baseline) to 32.0% (D30), and subsequently to 39.6% (D60). Elastography images at baseline (E) and D60 post‐treatment (F); clinical image before (G) and after treatment (H). In the elastograms, the grayscale displays the anatomical structures, while the colored overlays represent tissue elasticity through strain elastography. S (Soft): Areas with greater deformation (softer tissue), shown in warm colors (red, orange, and yellow), H (Hard): Areas with minimal deformation (stiffer tissue), shown in cool colors (green and blue).

Strain elastography revealed areas of increased stiffness in the lower and middle thirds of the face at baseline, whereas a reduction in tissue stiffness after treatment (Figure 3). The lower the stiffness the smoother the skin.

### 3.4 Investigator Reported Outcomes (GAIS Scores)

The investigator reported “much improved” or “very much improved” outcomes in 100% of single‐treatment patients and all two‐treatment cases corroborating patient's ratings after single‐treatment and two‐treatment.

### 3.5 Patient Reported Outcomes

All patients expressed high levels of satisfaction with the procedure, particularly appreciating the comfort and quick recovery time associated with the microcannula. All patients reported an improvement in skin texture and were at least “satisfied”, with 85.0% (*n* = 34/40) being “very satisfied” at the end of the trial.

More than 83% of the patients (*n* = 5/6) with rosacea reported reduced redness and inflammation, while 83.3% (*n* = 10/12) of patients with acne‐prone skin reported noticeable reduction in inflammation or erythema. Sixty‐two percent of the patients (*n* = 25/40) reported improved skin hydration as the most improved aspect after treatment. No statistical significance was observed between single or 2 treatments concerning patient GAIS scores and VAS pain score.

### 3.6 Safety

No serious AEs were reported. Minor and transient side effects, such as mild erythema at the injection site and ecchymosis, were reported in 15.0% (*n* = 6/40) and 7.5% (*n* = 3/40) of the patients respectively and resolved spontaneously within 24–72 h. Patients reported minimal discomfort, with 75.0% (*n* = 30/40) of the patients reporting an average VAS pain score of 1 (out of 10).

## 4 Discussion

With aging process, skin soft tissue physiology becomes compromised due to reduced collagen and elastin content synthesis, a decline in fibroblast density, and increased ECM degradation mediated by up‐regulated matrix metalloproteinases (MMPs) [4, 6]. Elastin presents a higher degree of calcification and degradation [7] compared to non‐aged skin, whilst decline in HA concentration results in reduced elasticity and ability to hold water in skin, thereby decreasing the volume of the dermis, and increasing the tendency to wrinkle [8]. Loss of dermal elasticity is a hallmark of early onset of photo‐damaged skin [3].

Different approaches to improve and rejuvenate skin physiology have been described in the literature. Superficial (intra‐ and/or subdermal) injections of lightly, or minimally, crosslinked HA (skin boosters) over a larger area is a relatively novel approach. HA is a negatively charged disaccharide unit, and multiple HA disaccharides in HA polymers give rise to a highly hydrophilic chain which strongly attracts water. Upon injections, this osmotic effect of HA gels creates a swelling pressure or turgor that enables the ECM to withstand compressive forces. Moreover, HA impacts skin turgor, cell integrity, mobility, and proliferation [9].

This paper focused on CPM‐R, a skin booster which has a polydensified structure created a two‐stage CPM‐HA crosslinking technology [10, 11]. The resulting gel is highly cohesive and capable of seamlessly integrating into the dermal tissue, filling spaces between collagen bundles and elastin fibrils, mimicking natural HA. CPM‐R is non animal derived gel cross‐linked with 1,4‐butanediol diglycidyl ether (BDDE), and contains 20 mg/mL HA with 17.5 mg/mL glycerol of nonanimal origin [12]. Subdermal injections of CPM‐R function as dermal hydration “microreservoirs” by drawing interstitial fluid into the dermal ECM, which helps to improve cell turgor and tissue tension without building up much volume. Additionally, CPM‐R impacts physiological processes in the ECM and stimulates dermal fibroblasts leading to collagen synthesis [12]. The hydrating effects of CPM‐R are long‐lasting, and may be related to the addition of glycerol, a highly hygroscopic tri‐hydroxy alcohol, which plays a central role in enhancing intrinsic moisture retention and ECM hydration dynamics [3]. Besides amplifying the hydrophilic nature of HA, glycerol has also been linked to improvement in stratum corneum integrity, barrier repair mechanisms, and reduction in trans‐epidermal water loss (TEWL), indicating that glycerol may be playing a therapeutic as well as cosmetic role, especially for patients with compromised skin barriers and chronic inflammatory states.

In the current study, 83% of patients with rosacea and 83.3% of acne‐prone patients reported improvement of skin redness or inflammation signs after CPM‐R injection, corroborating previously reported findings of significant reduction of skin redness, as well significant increase in gross elasticity, tone, radiance and hydration [3]. Improved viscoelastic properties, skin hydration and aesthetic appearance were observed in both the single‐treatment and two‐treatment groups with CPM‐R, consistent with outcomes reported in previous studies [12]. In published studies comparing CPM‐R to other HA‐based skin revitalization products, CPM‐R demonstrated superior hydration and elasticity improvement [13].

Mean increase in hydration levels compared to baseline was significantly higher for group 2 (two‐treatment) compared to group 1 (single treatment group). Within Group 2, the cumulative effect was perceived; hydration levels were significantly greater after the second treatment, indicating sustained effect of hydration, as well as booster effect, further supporting the need of a protocol of bi‐monthly treatments for optimal and sustained outcomes in patients with worse hydration levels at baseline.

There was a significant improvement in GAIS scores after CPM‐R treatment compared to baseline, as rated by both investigator and patients. For both group 1 and 2, overall satisfaction and GAIS scores were high following CPM‐R treatment, corroborating objective metrics such as corneometric hydration, which was significantly improved.

Corneometry and SE provide a more objective, quantitative assessment improving evaluation reliability. Ultrasonographic SE consists in a non‐invasive diagnostic technique used to quantify tissue stiffness independently of acoustic impedance and blood perfusion [4]. The lower the stiffness observed by SE, the smoother the tissue. We observed a significant reduction in stiffness in both single and two‐sessions cohorts, which further supports the high level of patient and investigator satisfaction in post‐treatment GAIS assessment (100% at least much improved). In dermatology, elastography has traditionally faced challenges due to the thin and layered structure of the skin, as well as the risk of measurement artifacts from adjacent bone or subcutaneous fat. However, advances in high‐frequency transducers have improved the resolution and applicability of elastography for facial skin analysis. Currently, there are no standardized visual or clinical scales that support SE as a validated method for assessing skin quality or elasticity.

This study represents an initial step toward exploring its potential application in dermatologic and aesthetic medicine. These preliminary findings highlight the need for further research to establish objective criteria and develop new evaluation tools for skin quality evaluation, opening a novel and promising field yet to be fully discovered.

Although the CPM‐R formulation does not contain lidocaine, patient‐reported pain scores remained low (1–2 out of 10)—the latter result was primarily attributed to the use of microcannula. The cannula‐delivery technique is believed to enable more homogeneous product distribution, reduce the risk of vascular injury and bruising, and facilitate a time‐efficient, atraumatic injection process. Additionally, the minimized risk of post‐procedure inflammation and irritation, particularly in patients with sensitive or inflammatory‐prone skin such as rosacea or acne, may contribute to faster recovery times and higher patient satisfaction. The ease of application, especially with fewer entry points, also made the microcannula a practical and efficient tool for the clinician. As a result, patients experienced greater comfort and shorter recovery times. Compared to traditional needle‐based methods, the use of microcannula minimized trauma to surrounding tissues, with ecchymosis observed in only 7.5% of cases (*n* = 3/40).

The limitations of this study largely relate to its retrospective, non‐randomized design and small convenience population, which, for instance, resulted in baseline hydration for group 2 being lower since clinically skin dryness was more severe. Nonetheless, it can reflect clinical practice since the treatment plan for each patient should be customized according to the clinical assessment of skin dryness. Exploratory studies require follow‐up using studies with comparative designs conducted in a larger patient cohort for increased power. Further validation should be performed to confirm findings, compare with other HA formulations, and explore longitudinal efficacy over 6–12 months. Nevertheless, this study highlights the importance of objective quantitative measurements to evaluate efficacy and underscores the use of CPM‐R with microcannula delivery as an effective therapeutic option with a robust safety profile and high patient satisfaction for improvement of skin quality.

## 5 Conclusion

This study demonstrated that the use of CPM‐R delivered using a retrograde linear and fanning technique with microcannula is an innovative, effective, safe, and well‐tolerated intervention for improving skin hydration and overall dermal quality in patients with mild to moderate signs of aging. Objective evaluation methods such as corneometry and ultrasound strain elastography allowed for quantitative and qualitative analysis of hydration and viscoelastic properties of the skin, confirming significant improvements beyond subjective satisfaction alone.

## Author Contributions

The author confirms being the sole contributor to this work and has approved the final manuscript. The author agrees with the journal submission, affirms accountability for all aspects of the study, and meets authorship criteria according to the ICMJE guidelines.

## Ethics Statement

This study was conducted in accordance with the 1964 Helsinki declaration and its later amendments or comparable ethical standards. A centralized institutional review board approval was obtained (Hospital La Misión, approval #IRB00011076). All patients have been informed and have provided written informed consent as well as photographic informed consent to have their anonymized data and related images to be published in this article.

## Conflicts of Interest

Dr. Suhail Luna is a consultant and Speaker for Merz Aesthetics Mexico.

## Data Availability

The data that support the findings of this study are available on request from the corresponding author. The data are not publicly available due to privacy or ethical restrictions.
